# Surface-Controlled Molecular Self-Alignment in Polymer Actuators for Flexible Microrobot Applications

**DOI:** 10.3390/polym11040736

**Published:** 2019-04-23

**Authors:** Minsu Jang, Jun Sik Kim, Ji-Hun Kim, Do Hyun Bae, Min Jun Kim, Donghee Son, Yong-Tae Kim, Soong Ho Um, Yong Ho Kim, Jinseok Kim

**Affiliations:** 1 Center for Bionics, Korea Institute of Science and Technology, Seoul 02792, Korea; minsujang@kist.re.kr (M.J.); kjs414@kist.re.kr (J.S.K.); daniel3600@kist.re.kr (D.S.); 2 School of Chemical Engineering, Sungkyunkwan University, Suwon 16419, Korea; 3 School of Electrical Engineering, Korea University, Seoul 02841, Korea; 4 SKKU Advanced Institute of Nanotechnology (SAINT), Sungkyunkwan University, Suwon 16419, Korea; kprhan@skku.edu (J.-H.K.); tanatos@skku.edu (Y.-T.K.); 5 Department of Biomedical Engineering, Sungkyunkwan University, Suwon 16419, Korea; bdh2568@skku.edu; 6 Department of Mechanical Engineering, Southern Methodist University, Dallas, TX 75275, USA; mjkim@lyle.smu.edu

**Keywords:** polymer actuator, self-alignment, azobenzene, microelectromechanical systems

## Abstract

Polymer actuators are important components in lab-on-a-chip and micromechanical systems because of the inherent properties that result from their large and fast mechanical responses induced by molecular-level deformations (e.g., isomerization). They typically exhibit bending movements via asymmetric contraction or expansion with respect to changes in environmental conditions. To enhance the mechanical properties of actuators, a strain gradient should be introduced by regulating the molecular alignment; however, the miniaturization of polymer actuators for microscale systems has raised concerns regarding the complexity of such molecular control. Herein, a novel method for the fabrication of micro-actuators using a simple molecular self-alignment method is presented. Amphiphilic molecules that consist of azobenzene mesogens were located between the hydrophilic and hydrophobic surfaces, which resulted in a splayed alignment. Thereafter, molecular isomerization on the surface induced a large strain gradient and bending movement of the actuator under ultraviolet-light irradiation. Moreover, the microelectromechanical systems allowed for the variation of the actuator size below the micron scale. The mechanical properties of the fabricated actuators such as the bending direction, maximum angle, and response time were evaluated with respect to their thicknesses and lengths. The derivatives of the polymer actuator microstructure may contribute to the development of novel applications in the micro-robotics field.

## 1. Introduction

The miniaturization of polymer actuators, which can be controlled through specific motions such as moving and grasping via external stimuli, is a critical factor in the control of micron-sized objects for diagnostics, drug delivery, surgery, and cellular manipulation [1,2,3]. Actuators can operate in response to various stimuli such as ion concentration gradients, heat, electrical fields, and light [4,5]. Among them, light offers several advantages for a wide range of applications, as it allows for the operation of the actuator in air and solutions using wireless, prompt, and target stimuli. Moreover, the variation in its wavelength and intensity can result in different actuator movements.

Photochromic molecules such as azobenzene mesogens respond to light stimuli via photomechanical reactions within their chromophores [6]. The conformational changes of azobenzene mesogens are induced by trans–cis isomerization under ultraviolet (UV) and visible light irradiation, which consequently leads to a change in the molecular volume. Their incorporation and specific ordering in a polymer film can create gradients of molecular volume in its bulk [7]. Even if the variation of the molecular volume is modest, it can generate out-of-plane deformations via accumulation throughout the bulk. Meanwhile, the photoinduced deformation of polymer actuators containing azobenzene mesogens can also be obtained by the photoinduced phase transition between LC and the isotropic state [8]. Regardless of the mechanism of deformation, it can successively lead to contraction or expansion depending on the alignment of the molecules that contain azobenzene mesogens [9,10].

Although polymer films that contain azobenzene molecules have recently been investigated in relation to the use of their deformation properties in various applications such as light-driven plastic motors, inchworm walkers, and robot arms, all of these applications are at the sub-millimeter scale [11,12,13]. To create a specific order of azobenzene mesogens, previous studies have focused on film fabrication with a rubbed polyimide layer. However, this method is unsuitable for microscale products. This limitation can be overcome by using inkjet printing technology, and reducing the film dimensions to 100 μm in width and 500 μm in length [14]. However, the viscosity of the melted polymers, the monomer de-wetting on the surface, and the nozzle size limit the miniaturization of actuators. Another study fabricated a fiber-type actuator with an approximate diameter of 300 μm by post-cross-linking a series of copolymers [15]. However, the bending direction was determined as one-way, either toward or away from a light source, due to the cylindrically symmetric structure of the fibers.

In this paper, a novel method was proposed for the fabrication of micro-actuators that contain azobenzene polymer films. This was achieved by using molecular self-alignment through the interaction force between their polarity and the surface hydrophilicity, in addition to the molding process using microelectromechanical systems (MEMSs). A reduction in the actuator size to several tens of micrometers was achieved. In addition, for products with a photoinduced bending ability in opposite directions depending on the incident light direction, the molecules were self-aligned by modulating the hydrophilicity of the substrates. Finally, the maximum bending angle was evaluated with respect to the lengths and thicknesses of the fabricated micro-actuators.

## 2. Materials and Methods

### 2.1. Synthesis of Azobenzene Monomers

**Figure 1:**
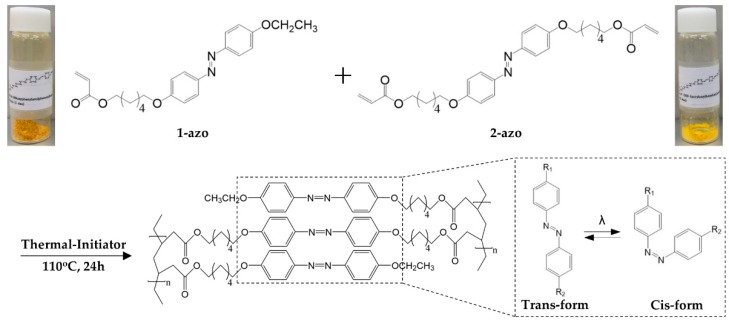
Chemical structures of the azobenzene liquid crystalline monomers (1-azo and 2-azo) used in this study and their polymerization schemes (the isomerization of the azobenzene group under ultraviolet light irradiation is indicated by the right dotted line).

The monomers of the azobenzene-based actuator, which include the monoacrylate 6-[4-(4-ethoxyphenylazo)-phenoxy]hexyl acrylate (1-azo) and diacrylate crosslinkers, in addition to 4,4′-di(6-acryloxy)hexyloxy azobenzene (2-azo) (Figure 1), were synthesized as described in the literature [16] and the Supplementary Materials. These monomers can be isomerized using a specific light wavelength.

### 2.2. Fabrication of Mold and Miniaturized Azobenzene Actuator

**Figure 2:**
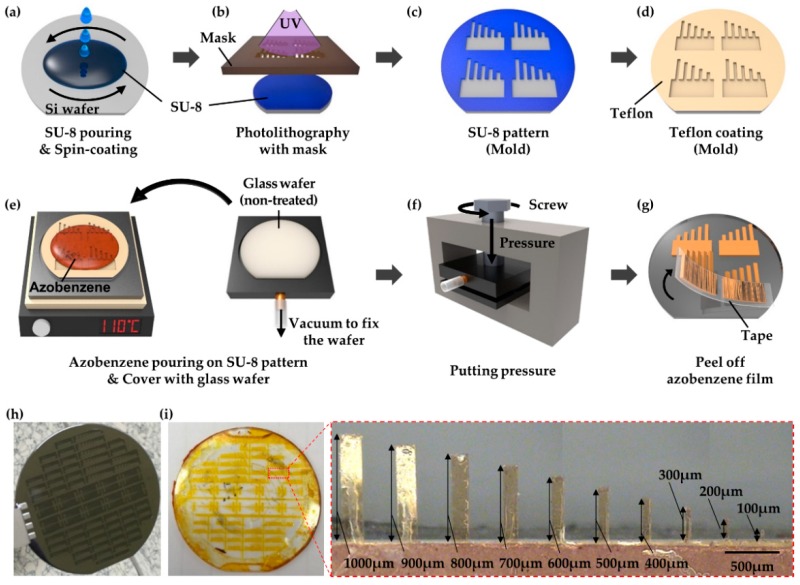
Fabrication process of tazobenzene actuators: (**a**–**d**) micron-sized mold with microelectromechanical system process, (**e**,**f**) monomer pouring and polymerization, and (**g**) actuator detachment. Fabrication results of (**h**) the mold on a silicon wafer and (**i**) the polymer actuators. The red box presents an image of the micro-actuators.

The manufacture of an azobenzene actuator consists of three main steps: mold fabrication on wafers using lithographic techniques (Figure 2a–d), monomer and pressure loading on the mold (Figure 2e,f), and polymerization (Figure 2g).

First, the mold was fabricated using silicon and glass wafers with diameters of 50.8 mm, which were preliminarily cleaned in a piranha solution (hydrogen peroxide and sulfuric acid in a 3:1 volume ratio). The silicon wafers were then immersed in a buffered oxide etchant to make their surfaces hydrophobic. Thereafter, they were further cleaned, and the negative photoresist SU-8 (MicroChem Corp., MA, USA) was patterned onto them using photolithography. Given that this technique requires that UV light passes through a photomask (Figure 2b), the pattern on the photomask determines the conformation of both the mold and actuator.

Various cantilever-type actuators of different lengths were fabricated simultaneously to evaluate the resultant bending motion under the same conditions. The actuators had a 5:1 aspect ratio with respect to the length and width in the ranges 100–1000 μm and 20–200 μm, respectively. In addition, molds with three different thicknesses (5 μm, 10 μm, and 20 μm) were prepared to investigate the correlation between the actuator thickness and bending properties under light irradiation. The patterning conditions, which include the SU-8 type, baking time, and UV exposure time, were varied based on the desired mold thickness. For example, on the 10-μm thick mold, SU-8 3010 was spin-coated at 3000 rpm for 30 s and soft baked at 65 °C for 90 s, and then at 95 °C for 450 s. The photoresist was successively exposed to UV light with an energy of 150 mJ/cm^2^; heated further at 65 °C for 90 s, and then at 95 °C for 4 min for post-exposure baking. To remove un-crosslinked SU-8, the wafer was immersed in an SU-8 developer for 3 min, and then rinsed with isopropyl alcohol and distilled water. After the fabrication, the thickness was checked using an Alpha-Step profilometer. The molds were coated with polytetrafluoroethylene for hydrophobicity, whereas the glass wafer substrates were only washed with a piranha solution to further enhance their hydrophilicity.

The monomer solutions were prepared by adding 1,1′-azobis(cyclohexane-1-carbonitrile) (2 mol %) as a thermal initiator to the mixtures of 1-azo and 2-azo (both 49 mol%). The polymerizable mixtures were loaded on the silicon molds and heated to 110 °C. The melted mixtures were then spread evenly on the substrate and maintained at 110 °C in the oven for 10 min to eliminate air pockets. Thereafter, the glass wafers were set on silicon molds, and their piles were placed in a pressure jig. Thermal polymerization was carried out by incubating the pressure jig in the oven at 110 °C for 24 h. Finally, the polymerized actuator films were detached from the substrates using tape.

Furthermore, to evaluate the polymer alignment, the fracture surfaces were evaluated using a field emission scanning electron microscope (FESEM) (Hitachi S-4200, Tokyo, Japan) that was operated at 15 kV in a high vacuum environment. The samples were frozen in liquid nitrogen, broken to expose polymer cross-sections, and then coated with a 10-nm thick Pt layer. In addition, we measured the polarized attenuated total reflectance (ATR) infrared spectra of the film at room temperature with a Fourier-transform infrared (FTIR) spectrometer (FT/IR-4100, Jasco, MD, USA).

### 2.3. Characterization of Photoinduced Bending Behavior

To evaluate the bending motion of the actuators under incident light, fabricated cantilevers were mounted on slide glasses with their tips in air and irradiated at room temperature at an intensity of 45 mW/cm^2^ using an Omnicure S2000 (Excelitas Technologies Corp., MA, USA) with a 365-nm band-pass filter and a collimator to ensure uniform intensity throughout the cantilever tip.

The bending motion was monitored using a charge-coupled device (CCD) camera. The bending angle, which was obtained using ImageJ software (Version 1.52a, NIH, MD, USA), was defined as the angular variation of the line that connected the first cantilever region to the tip.

## 3. Results

Micron-sized molds fabricated via the microelectromechanical systems (MEMS) process were used for the actuator miniaturization (Figure 2h). The inherent benefit of the MEMS allowed for several samples to be obtained simultaneously (Figure 2i). The ratio of the monomers in the mixtures of 1-azo, 2-azo, and the thermal initiator, which were loaded on the molds to obtain cantilever-type actuators, was a critical factor. For example, if the amount of 2-azo was significantly lower than that of 1-azo (e.g., 1-azo:2-azo = 7 mol%:3 mol%), it could result in a powder product. Moreover, 2-azo, which plays a role as a crosslinking agent, should be evenly distributed to link the 1-azo monomers throughout the mold surface.

**Figure 3:**
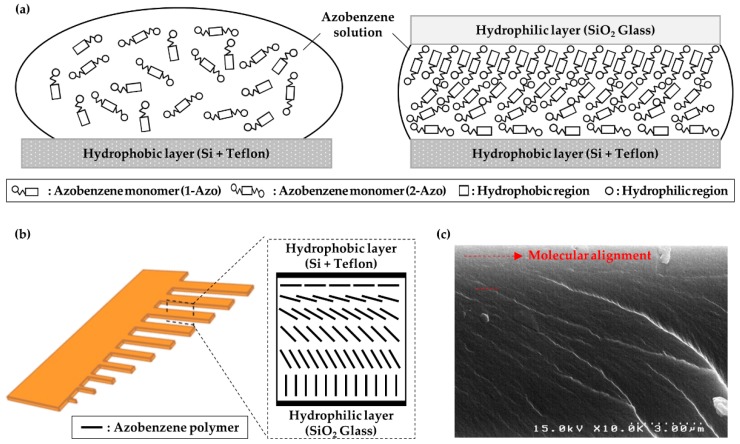
(**a**) Schematics of the interaction between amphiphilic azobenzene monomers and mold surface; (**b**) molecular orientation of the polymerized actuator; and (**c**) cross-sectional scanning electron microscopy image of the actuator; the fracture shows the molecular alignment (red dotted arrow).

Many actuators incorporate two or more materials in a bilayer structure and use the contraction or extension of one layer for bending movements [17,18]. However, although the fabricated azobenzene-based actuators consisted of a single layer, they could also bend in a direction due to the variation in the inner molecular orientation, which was dependent on the layer depth. The molecules could self-align by the interaction between the substrate and monomers. The lower mold surface was coated with Teflon and was therefore hydrophobic, whereas the piranha solution, which is a strong oxidizing agent, enhanced the hydrophilicity of the glass mold surface by removing the organic residues and by hydroxylation (Figure 3a). In addition, the molecular structure of the azobenzene monomer consisted of two parts: two phenyl rings at the center that make it non-polar, and a polar ester group at its end. Due to the interaction between the surface hydrophilicity and the polarity of the molecule, the molecular orientation of the first layer on the hydrophobic surface was parallel, whereas that of the first layer on the hydrophilic surface was relatively perpendicular. The molecules in the first layer could orient their neighboring molecules and thus determine the alignment of adjacent molecules [19,20,21,22]. Consequently, a splayed alignment was formed through the actuator depth (Figure 3b), as revealed by the variation in the orientation of the polymerized molecules in the cross-sectional SEM image of the azobenzene film presented in Figure 3c. In addition, we measured both surfaces by polarized ATR infrared spectroscopy to clarify the molecular alignment (Supplementary Materials, Figure S2). The peaks at 1597 and 1499 cm^−1^ are characteristic of the stretching vibration of phenyl rings linked by an N=N double bond. The result showed that the transmittance of azobenzene mesogens in the surface in contact with the hydrophilic substrate was more than that in the surface contact with the hydrophobic surface. It is well known that the greatest amount of the polarized IR light beam can be transmitted once the alignment of mesogens is vertical to the direction of the light. However, the minimum transmittance is obtained when two directions are parallel to each other. Therefore, the molecular alignment in contact with the glass substrate is perpendicular to the surface while the molecules in contact with the silicon substrate are aligned along the surface. Moreover, this phenomenon has an influence on the bending direction of cantilever-type actuators.

Prior to the evaluation of the bending properties, the wavelength that would be absorbed by the azobenzene moieties was identified by carrying out UV–visible light absorption measurements (Supplementary Materials, Figure S3). The actuators absorbed wavelengths in the range 300–500 nm. In particular, the maximum absorbance was observed for UV-A due to the π–π* absorption associated with the trans isomer. Based on the results, the absorbances of the fabricated actuators were compared before and after irradiation using light with a wavelength of 365 nm for 5 s. Prior to irradiation, the absorbance level at 480 nm was lower than that at 365 nm, which indicated that the film was mainly composed of the trans isomer. However, after irradiation, this was changed into a cis isomer by the π–π* absorption, as revealed by a significant absorption increase at 480 nm and decrease at 365 nm.

**Figure 4:**
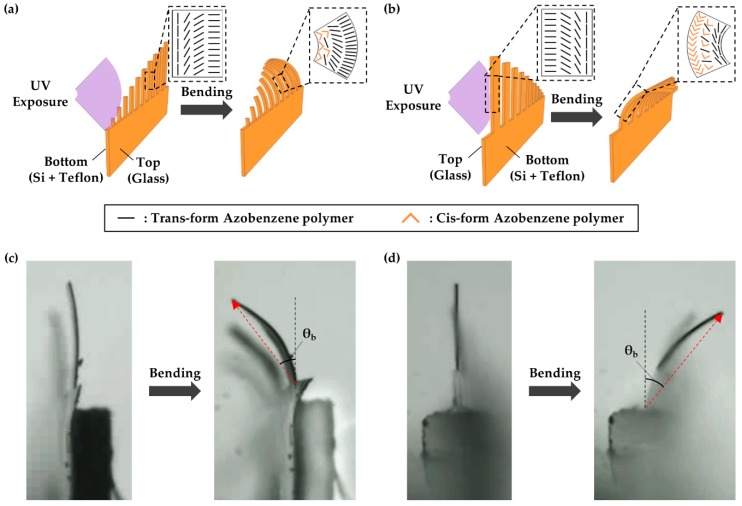
The relationship between the alignment of illuminated molecules and the bending direction by isomerization; the light exposure to (**a**,**c**) homogeneous and (**b**,**d**) homeotropic surfaces (depending on whether the surface faces the incident light, the bending direction could be the opposite).

As above-mentioned, the molecular orientation differed on the two sides of the actuators. This property significantly influences the bending direction. In particular, depending on the orientation, the trans–cis isomerization of the azobenzene moieties can induce a change in the molecular volume, thus providing the driving force for the contraction or expansion of the actuator. In addition, the strong π–π* absorption can prevent UV light penetration through the film depth until the majority of the trans isomers on the surface have been transformed into cis isomers. When the surface that had a homogeneous alignment with molecules ordered parallel to the surface faced toward the incident UV light, the actuator bent along the direction of exposure (Figure 4a,c), since the polymer occupied a smaller area when the trans isomers transformed into cis isomers. In contrast, the homeotropic alignment in the surface induced expansion due to the increase in the molecular volume, which caused the actuator to bend in the reverse bending direction (Figure 4b,d).

**Figure 5:**
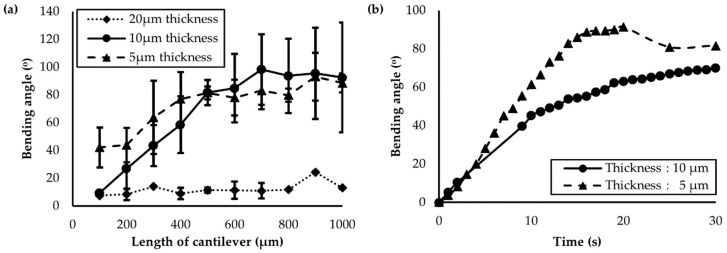
(**a**) Maximum bending angle as a function of actuator length and thickness; (**b**) change in the bending angle with respect to the ultraviolet exposure time. Actuators with thicknesses of 10 μm and 5 μm (solid and dashed lines, respectively) were compared.

The influence of actuator length and thickness on the bending characteristics were evaluated by illuminating the side with the homogeneous alignment using light with a wavelength of 365 nm and intensity of 45 mW/cm^2^. Figure 5a presents the maximum bending angles after irradiation for actuators of different lengths. The thicker actuators (20 μm) exhibited a maximum bending angle of only 20°, whereas those with thicknesses of 5 μm and 10 μm had bending angles greater than 90°. The greater bending angles reached by the thinner actuators may be because the bending was induced by the surface strain from the variation of the molecular volume.

Although the bending angle was different with respect to the actuator thickness and length; it increased proportionally in accordance with an increase in length, irrespective of the thickness when it was below 90°. The proportional increase indicates that the bending curvature had the same value. The curvature of the actuators with thicknesses of 10 μm was 0.052 from a length of 100–600 μm. Given that the concentration of the azobenzene moieties on the surface, which could absorb all the initial UV light, in addition to the impact on the bending, was consistent along the actuator surfaces; the bending per unit area was constant. However, a linear increase was not observed for bending angles greater than 90°. One explanation for this may be that the opposite side of the actuators that expand under UV illumination could be exposed to the light, which may have an influence on the bending angle.

To evaluate the bending properties of the actuators with respect to the exposure time, actuators with lengths of 400 μm and thicknesses of 5 μm and 10 μm were illuminated for 30 s (Figure 5b). Irrespective of the thickness, the maximum bending angles were 90° and 60° for the actuators with thicknesses of 5 μm and 10 μm, respectively, over a time-period of approximately 15 s. This is because the number of azobenzene moieties located on the surface, which directly influences the bending angle, was proportional to the surface area. Therefore, the time required for isomerization was the same.

## 4. Conclusions

In this study, micron-sized light-driven actuators were successfully fabricated via thermo-initiated polymerization with molds obtained by the MEMS process. The polymerized molecules were self-aligned as parallel or perpendicular to the surface of the actuators. This alignment was induced by the interaction between the polarity of the amphiphilic molecules and the hydrophilic/hydrophobic properties of the mold surfaces. The splayed (homogeneous and homeotropic) alignment was confirmed by SEM imaging; thus, the opposite bending direction was observed according to the irradiated surface. The actuator bent toward the light when the homogeneous surface was irradiated, and in the opposite direction in the case of the homeotropic surface. This indicates that the homogeneous surface contracts and the homeotropic surface expands under UV irradiation. The bending behavior of the fabricated actuators varied with respect to their lengths and thicknesses. In particular, the longer and thinner actuators exhibited larger bending angles. However, the maximum bending angle was reached quickly, irrespective of the actuator thickness. Thereafter, the actuators gradually bent toward their original positions under continuous irradiation due to the reduction in the strain gradient.

The proposed method for the fabrication of micro-actuators with self-alignment and their fast and large photomechanical response could contribute to the development of applications such as wireless microrobots based on UV light irradiation without requiring conventional robot components such as batteries and gears.

## Supplementary Materials

The following are available online at https://www.mdpi.com/2073-4360/11/4/736/s1.

## References

[1] Camacho-Lopez M., Finkelmann H., Palffy-Muhoray P., Shelley M. (2004). Fast liquid-crystal elastomer swims into the dark. Nat. Mater..

[2] Jager E.W., Smela E., Inganäs O. (2000). Microfabricating conjugated polymer actuators. Science.

[3] Jager E.W.H., Ingana O. (2000). Microrobots for Micrometer-Size Objects in Aqueous Media: Potential Tools for Single-Cell Manipulation. Science.

[4] Sawa Y., Urayama K., Takigawa T., Desimone A., Teresi L. (2010). Thermally driven giant bending of liquid crystal elastomer films with hybrid alignment. Macromolecules.

[5] Shintake J., Rosset S., Schubert B., Floreano D., Shea H. (2016). Versatile Soft Grippers with Intrinsic Electroadhesion Based on Multifunctional Polymer Actuators. Adv. Mater..

[6] Yu Y., Nakano M., Ikeda T. (2003). Directed bending of a polymer film by light. Nature.

[7] Yu Y., Nakano M., Shishido A., Shiono T., Ikeda T. (2004). Effect of Cross-linking Density on Photoinduced Bending Behavior of Oriented Liquid-Crystalline Network Films Containing Azobenzene. Chem. Mater..

[8] Yu H., Ikeda T. (2011). Photocontrollable liquid-crystalline actuators. Adv. Mater..

[9] Van Oosten C.L., Harris K.D., Bastiaansen C.W.M., Broer D.J. (2007). Glassy photomechanical liquid-crystal network actuators for microscale devices. Eur. Phys. J. E.

[10] Kondo M., Yu Y., Ikeda T. (2006). How does the initial alignment of mesogens affect the photoinduced bending behavior of liquid-crystalline elastomers?. Angew. Chem. Int. Ed..

[11] Yamada M., Kondo M., Mamiya J.I., Yu Y., Kinoshita M., Barrett C.J., Ikeda T. (2008). Photomobile polymer materials: Towards light-driven plastic motors. Angew. Chem. Int. Ed..

[12] Yamada M., Kondo M., Miyasato R., Naka Y., Mamiya J., Kinoshita M., Shishido A., Yu Y., Barrett C.J., Ikeda T. (2009). Photomobile polymer Materials—Various Three-dimensional movements. J. Mater. Chem..

[13] Cheng F., Yin R., Zhang Y., Yen C.-C., Yu Y. (2010). Fully plastic microrobots which manipulate objects using only visible light. Soft Matter.

[14] van Oosten C.L., Bastiaansen C.W.M., Broer D.J. (2009). Printed artificial cilia from liquid-crystal network actuators modularly driven by light. Nat. Mater..

[15] Cheng Z., Ma S., Zhang Y., Huang S., Chen Y., Yu H. (2017). Photomechanical Motion of Liquid-Crystalline Fibers Bending Away from a Light Source. Macromolecules.

[16] Li C., Lo C.W., Zhu D., Li C., Liu Y., Jiang H. (2009). Synthesis of a photoresponsive liquid-crystalline polymer containing azobenzene. Macromol. Rapid Commun..

[17] Hu J., Li X., Ni Y., Ma S., Yu H. (2018). A programmable and biomimetic photo-actuator: A composite of a photo-liquefiable azobenzene derivative and commercial plastic film. J. Mater. Chem. C.

[18] Ma S., Li X., Huang S., Hu J., Yu H. (2019). A Light-Activated Polymer Composite Enables On-Demand Photocontrolled Motion: Transportation at the Liquid/Air Interface. Angew. Chem. Int. Ed..

[19] Tiwari A.K., Pattelli L., Torre R., Wiersma D. Remote Control of Liquid Crystal Elastomer Random Laser. Proceedings of the 2017 IEEE Workshop on Recent Advances in Photonics (WRAP).

[20] Priimagi A., Barrett C.J., Shishido A. (2014). Recent twists in photoactuation and photoalignment control. J. Mater. Chem. C.

[21] Seki T., Fukuda R., Tamaki T., Ichimura K. (1994). Alignment photoregulation of liquid crystals on precisely area controlled azobenzene Langmuir-Blodgett monolayers. Thin Solid Films.

[22] Lee S.H., Yoon T.H., Kim J.C., Lee G.D. (2006). Reverse tilt domains in liquid crystal cells with a splayed director configuration. J. Appl. Phys..

