# Supplementary material for: Surface-Controlled Molecular Self-Alignment in Polymer Actuators for Flexible Microrobot Applications
Source: Polymers (Basel). 2019 Apr 23;11(4):736. doi: 10.3390/polym11040736 (PMC6523470; doi:10.3390/polym11040736)
Supplement: Supplementary file 1 [file polymers-11-00736-s001.pdf]

Supplementary Materials

Surface-Controlled Molecular Self-Alignment in Polymer Actuators for Flexible Microrobot Applications

Minsu Jang [1,2,†], Jun Sik Kim [1,3,†], Ji-Hun Kim [4], Do Hyun Bae [5], Min Jun Kim [6], Donghee Son [1], Yong-Tae Kim [4], Soong Ho Um [2,4,*], Yong Ho Kim [4,5,*], Jinseok Kim [1,*]

1 Center for Bionics, Korea Institute of Science and Technology, Seoul 02792, South Korea; minsujang@kist.re.kr (M.J.), kjs414@kist.re.kr (J.S.K.), daniel3600@kist.re.kr (D.S.)
2 School of Chemical Engineering, Sungkyunkwan University, Suwon 16419, South Korea
3 School of Electrical Engineering, Korea University, Seoul 02841, South Korea
4 SKKU Advanced Institute of Nanotechnology (SAINT), Sungkyunkwan University, Suwon, 16419, South Korea; kprhan@skku.edu (J-H.K.), tanatos@skku.edu (Y-T.K.)
5 Department of Biomedical Engineering, Sungkyunkwan University, Suwon 16419, South Korea; bdh2568@skku.edu (D.H.B)
6 Department of Mechanical Engineering, Southern Methodist University, Dallas TX 75275, USA; mjkim@lyle.smu.edu (M.J.K)
* Correspondence: sh.um@skku.edu (S.H.U.), yhkim94@skku.edu (Y.H.K.), jinseok@kist.re.kr (J.K.); Tel.: +82-2-958-6745 (J.K.)
† These authors contributed equally to this work.

S1. Synthesis of 1-Azo and 2-Azo

S1.1. Synthesis of 1-Azo

First, p-phenetidine and sodium nitrite (20 mmol each) were dissolved in a 40 mL solution of water and ethanol (20 mL each). Under continuous stirring in an ice bath, a mixture of HCl (4.2 mL) and cold water (20 mL) was slowly added. The resulting solution was stirred for 90 min and the pH was adjusted to 1 by adding more HCl. After stirring for another 30 min, the resulting precipitate was separated by suction filtration, washed with excess water, and dried to obtain 4-hydroxy-4′-ethoxyazobenzene (Compound 2) with a yield of 81.5%.

Compound 2 (16.3 mmol), 6-chlorohexan-1-ol (19.6 mmol), and a catalytic amount of KI were dissolved in dimethyl formamide (DMF) (100 mL) and stirred at 120 °C for 6 h. The resulting mixture was transferred into excess water (about 1000 mL) and the precipitate was extracted with ethyl acetate. The organic layer was dried with $MgSO_4$ and the residual solvent was evaporated. Then, the precipitate was recrystallized with ethanol to obtain 4-(6-hydroxyhexyloxy)-4′-ethoxyazobenzene (Compound 3) with a yield of 69.3%.

Compound 3 (11.36 mmol), triethylamine (31.8 mmol), and a catalytic amount of hydroquinone were dissolved in tetrahydrofuran (THF) (30 mL). After cooling the solution down to 0 °C, acryloyl chloride (2.77 mL) was added dropwise under a nitrogen atmosphere. The mixture was stirred for 26 h at room temperature. Then, a supersaturated solution of $NaHCO_3$ was added and extracted with ethyl

acetate. The organic layer was dried with $MgSO_4$. After the solvent evaporation, the crude product was purified via column chromatography with chloroform to obtain 6-[4-(4-ethoxyphenylazo)phenoxy]hexyl acrylate (1-azo) with a yield of 73.0%.

S1.2. Synthesis of 2-Azo

First, 4,4′-dihydroxyazobenzene (10 mmol), K_2CO_3 (26 mmol), and a catalytic amount of KI were dissolved in DMF (15 mL). The solution was refluxed at 120 °C for 20 h. Then, excess water was added, and the precipitate was collected by suction filtration and dried. A solution of THF and chloroform (1:1 volume ratio) was added to extract the product. The organic layer was dried with $MgSO_4$ and the solvent was evaporated. The resulting precipitate was purified by recrystallization with a THF–chloroform solution to obtain 4,4′-bis(6-hydroxyhexyloxy)azobenzene (Compound 5) with a yield ratio of 53%.

Compound 5 (5.3 mmol), trimethylamine (5.3 mmol), and a catalytic amount of hydroquinone were dissolved in dehydrated THF (30 mL). The resulting solution was stirred and cooled down to 0 °C under a nitrogen atmosphere. Acryloyl chloride (18.55 mmol) was slowly dissolved into the solution, which was stirred for 4 h in an ice bath. Then, the temperature was adjusted to room temperature and the solution was further stirred for other 24 h. Excess water was added, and the precipitate was extracted with chloroform and dried with $MgSO_4$. The crude product was purified via column chromatography with a solution of ethyl acetate and chloroform (1:1 volume ratio). The resulting product was recrystallized with methanol to obtain 4,4′-di(6-acryloxy)hexyloxy azobenzene (2-azo) with a yield of 56.4%.

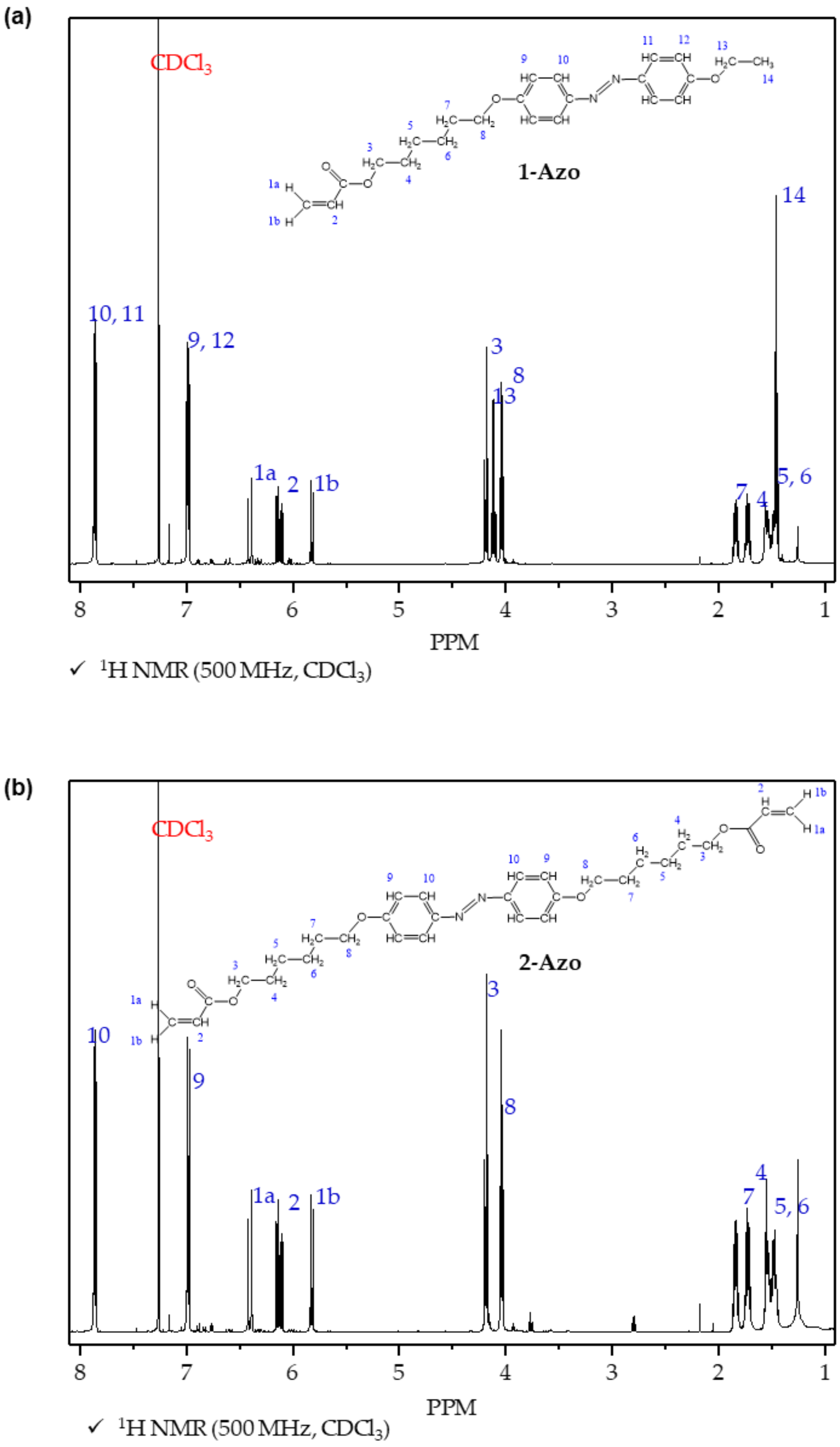

Figure S1. Confirmation of (**a**) 6-[4-(4-ethoxyphenlazo)phenoxy]hexyl acrylate (1-azo) and (**b**) 4,4′-di(6-acryloxy)-hexyloxy azobenzene (2-azo) by proton nuclear magnetic resonance spectra.

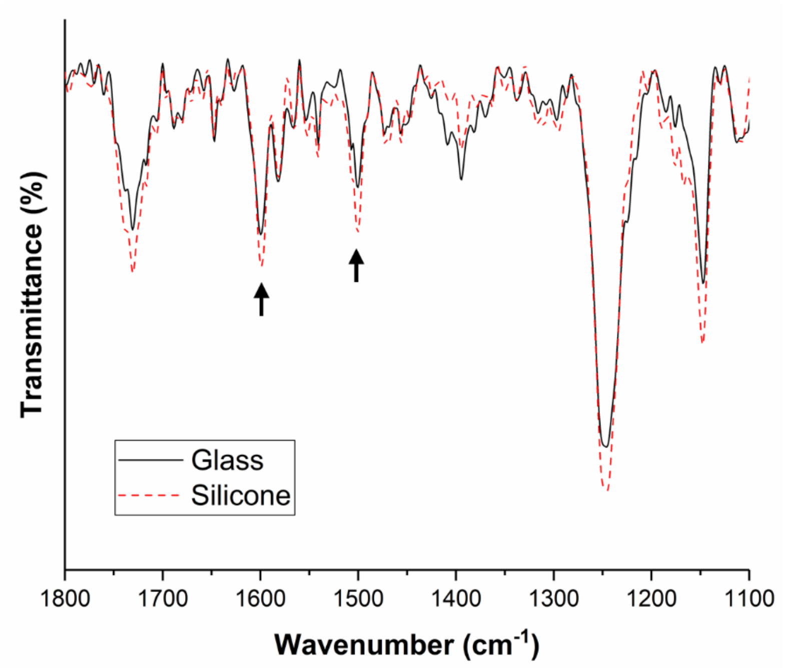

Figure S2. Polarized FTIR spectra of the surface in contact with the glass (black, solid line) and silicon (red, dashed line) substrate in the azobenzene actuator; the arrows indicate the peaks at 1597 and 1499 cm^{-1}, respectively.

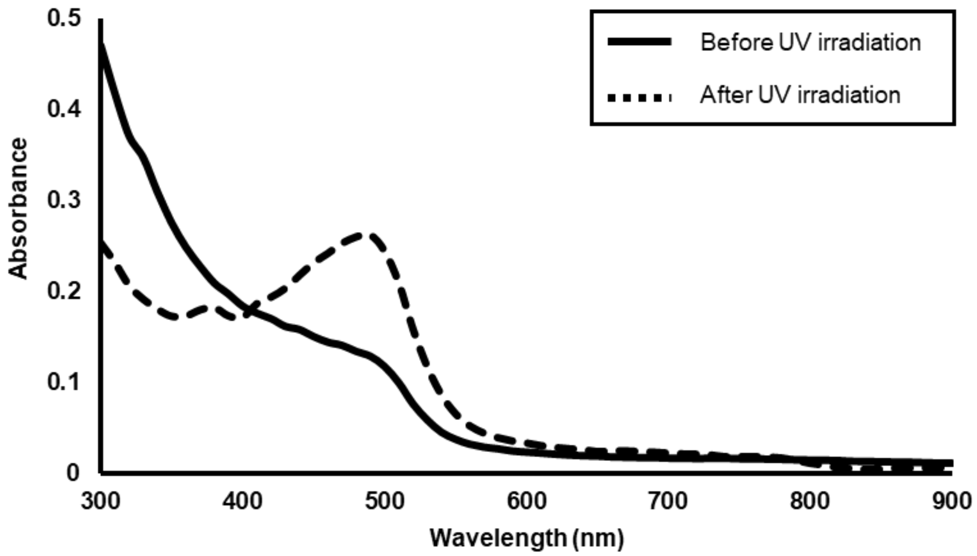

Figure S3. Absorbance properties of azobenzene-based actuators with a thickness of 5 μm.
